# InsiliCoil: An Integrated Software Suite for Coiled Coil Design, Prediction, and Therapeutic Engineering

**DOI:** 10.1021/acssynbio.5c00678

**Published:** 2025-12-10

**Authors:** Jaiveer Arora, Jody M. Mason

**Affiliations:** Department of Life Sciences, 1555 University of Bath, Bath BA2 7AY, U.K.

**Keywords:** coiled coil, protein−protein interactions, synthetic biology, computational design, orthogonal interactomes, bZIP, high-throughput in silico screening

## Abstract

Helical protein–protein interactions underpin transcriptional regulation, signal transduction, and self-assembly, yet their rational design remains challenging. Coiled coils (CCs) are particularly attractive as modular, programmable building blocks in synthetic biology, while also serving as therapeutic targets. Here we present **InsiliCoil**, a cross-platform software suite that unifies predictive modeling, selective peptide inhibitor discovery, and orthogonal interactome design into a single accessible framework. At its core, **isCAN** enables high-throughput identification of selective CC inhibitors, while **CCIS** systematically constructs orthogonal CC networks for synthetic biological circuits and biomaterials. Additional utilities support automatic heptad detection, heptad scanning, constraint analysis, charge block prediction, library generation, and large-scale visualization. Benchmarking against experimental data sets confirms that InsiliCoil reliably recovers validated inhibitors and interactomes, while offering orders-of-magnitude faster throughput than structure-based approaches. By providing a cohesive, user-friendly platform for controlling helix-mediated PPIs, InsiliCoil accelerates both therapeutic discovery and the rational engineering of programmable biological systems.

## Graphical abstract

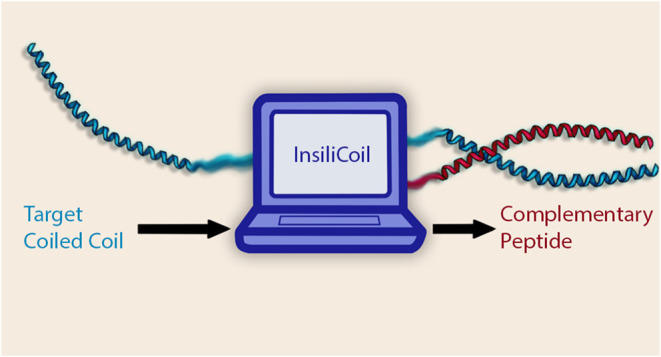

## Introduction

Protein–protein interactions (PPIs) drive essential biological processes such as signal transduction, DNA replication, and gene transcription.^1^ Among the many motifs mediating PPIs, coiled coils (CC) are especially prevalent, comprising 3–5% of all known protein structures.^2^ Defined by a repeating heptad pattern, designated [***abcdefg***]_n_, in which each position favors distinct physicochemical characteristics. These are most notably, hydrophobic residues at the ***a*** and ***d*** positions.^2–4^ Coiled coils offer an elegant balance of structural simplicity and functional specificity. This makes them ideal scaffolds for probing interaction principles and for engineering new protein–protein interfaces.

Decades of research have uncovered key principles governing CC stability, specificity, and oligomerization.^5–9^ These insights have enabled wide application in synthetic biology, where both natural and *de novo* designed CCs have been used to construct molecular scaffolds, programmable nanomaterials, self-assembling fibers, and components for bioelectronics.^10–19^

Beyond their bioengineering utility, CCs are also attractive therapeutic targets, particularly those within the basic leucine zipper (bZIP) family of transcription factors, which are implicated in diseases such as rheumatoid arthritis and cancer.^20–25^ Although numerous efforts have sought to inhibit dysfunctional CCs using small molecules,^26^ success has been limited; for example the bZIP inhibitor T-5224 progressed to Phase II clinical trials before being discontinued.^27,28^ This difficulty arises from the lack of well-defined binding pockets, rendering many CCs effectively ″undruggable″.^29^ Consequently, attention has shifted toward peptide-based inhibitors,^30–35^ which rely on designing stable, selective heterodimeric CCs that can outcompete native interactions.

Advances in computational biology have transformed the study of protein interactions and accelerated tool development across molecular design.^36–38^ In the CC space, numerous algorithms now support structure prediction,^39,40^ oligomeric state determination,^41^ and partner specificity analysis.^5,7,42–44^ Yet despite this expanding ecosystem, researchers still lack an accessible, integrated, and high-throughput platform for CC design. In particular one that enables seamless *in silico* prediction, visualization, and optimization without requiring coding expertise or complex setup.

The success of predictive frameworks like AlphaFold has highlighted the power of *in silico* screening for protein–protein interaction discovery.^45–47^ For instance, Homma et al. used AlphaFold2 and AlphaFold-Multimer to screen over 11,000 pathogen-host pairs, identifying 15 novel interactions, but at a cost exceeding 13,000 CPU hours and 8,000 GPU hours.^47,48^ While such methods offer remarkable structural precision, their computational demands render them impractical for large-scale or iterative design workflows in most research settings.

In contrast, CCs offer a uniquely tractable system for high-throughput simulation, owing to their well-characterized sequence–structure relationships. A prime example is the bZIP Coiled-coil Interaction Prediction Algorithm (bCIPA),^43,44^ which quantitatively predicts thermal stability (*T*_m_) from primary sequence alone. bCIPA integrates helical propensity (HP), core packing (C), and electrostatic interactions (ES) using the formula

1 $$T_{m}=a\times \mathrm{HP}+b\times C+c\times \mathrm{ES}+d$$

with empirically derived coefficients (*a* = 81.3256, *b* = −10.5716, *c* = −4.7771, *d* = −29.1320).^41,42^ Unlike qualitative scoring schemes, bCIPA enables direct comparison between designs and has been shown to classify 97% of strong interactions and 95% of noninteractions in benchmark data sets.^44,49^ Its predictive performance rivals data-driven models and often exceeds that of structure-based approaches.^50^

Several computational CC screens have been built around bCIPA,^8,49,51–53^ yet many suffer from limited accessibility, poor scalability, or unintuitive interfaces. These factors restrict widespread adoption and their use in high-throughput workflows. To address these challenges, we developed InsiliCoil, a fast, robust, and user-friendly software suite for Windows and macOS that enables comprehensive GUI-based CC research without the need for coding or complex software setup.

InsiliCoil includes two flagship tools: (1) *isCAN*, a high-throughput screening engine that identifies peptide inhibitors capable of forming selective heterodimeric CCs with a user-defined target while excluding homodimeric and off-target pairings; and (2) *Coiled Coil Interactome Screen* (CCIS) that identifies the largest orthogonally interacting set from a custom peptide library. The platform also offers modules for helical constraint prediction, charge block analysis, peptide library generation, and batch-mode visualization, including helical wheels and graphical flowcharts of interaction outcomes. Together, these components make InsiliCoil a powerful and accessible platform for accelerating therapeutic discovery and programmable CC design.

## Results

Coiled coils (CCs) are ubiquitous mediators of protein–protein interactions (PPIs), particularly within essential processes such as gene transcription. Despite their structural simplicity, CCs exhibit highly specific pairing behaviors and are increasingly implicated in disease. Their lack of well-defined binding pockets often renders them “undruggable” by small molecules. At the same time, their modularity, predictable geometry, and tunable specificity make CCs ideal scaffolds for bioengineering, smart materials, and therapeutic design, driving demand for tools that support rational CC design at scale.

To address the growing need for intuitive, high-throughput, and experimentally aligned computational tools in this space, we present InsiliCoil: a cross-platform software suite designed to accelerate all facets of CC research. InsiliCoil integrates ten key modules:

1. **isCAN** – a high-throughput virtual screening engine that simulates PCA^43^ and CANDI^6^ assays to identify CC inhibitors. It outputs melting temperatures (*T*_m_), DrawCoil-style helical wheels, and a ranked list of peptides predicted to bind a user-defined target (T) while discriminating against target and library homodimerization (LL and TT), and interactions with competing off-targets (LC and TC). Visual and numerical flow diagrams quantify binding outcomes across all LT, LL, TT, LC, and TC complexes. The “Consensus-Coil” feature highlights positional variability among hits to inform rational design.
2. **Coiled coil interactome screen** (**CCIS**)^8,51^ – identifies the largest set of orthogonally interacting CC pairs from a user-provided library, supporting synthetic biology applications.
3. **Predict Helical Constraints** – highlights peptide sequences likely to benefit from conformational stabilization,^54^ using literature-derived structural heuristics.
4. **Predict Charge Blocks** – identifies complementary electrostatic blocks predicted to enhance intermolecular attraction and intramolecular repulsion, thereby stabilizing dimer formation, as described by Crooks et al.^8^
5. **Create Library** – allows users to design peptide libraries by defining positional variability within a sequence template.
6. **Build Library** – the reverse of Create Library; extracts consensus motifs and maps tolerated or variable residues across a given peptide set.
7. **BatchCoil** – enables rapid batch generation of DrawCoil-style helical wheels for large-scale visualization.
8. **Consensus-Coil** – visualizes residue variability within a CC library using a DrawCoil-style helical wheel.
9. **Automatic Heptad Frame Determination** – automatically identifies the correct heptad register without requiring a user-defined starting position.
10. **Heptad Scanning** – within isCAN the software evaluates alternate heptad alignments and selects that which provides the most favorable Δ*T*_m_ against the target.

In addition to these core modules, InsiliCoil includes a curated sequence database containing all bZIP sequences^55^ and all basic helix–loop–helix-leucine zipper (bHLHZIP) sequences, which were compiled by alignment (Supporting Figure 1).

Benchmarking against published experimental data sets confirms that InsiliCoil reliably predicts both CC inhibitor-target interactions and orthogonal CC sets. By integrating design, screening, prediction, and visualization in a single user-friendly interface for Windows and macOS, InsiliCoil offers a practical and accessible solution, bridging the gap between therapeutic development and synthetic biology. Below we detail the performance and attributes of the ten integrated software packages (Figure 1).

**Figure 1:**
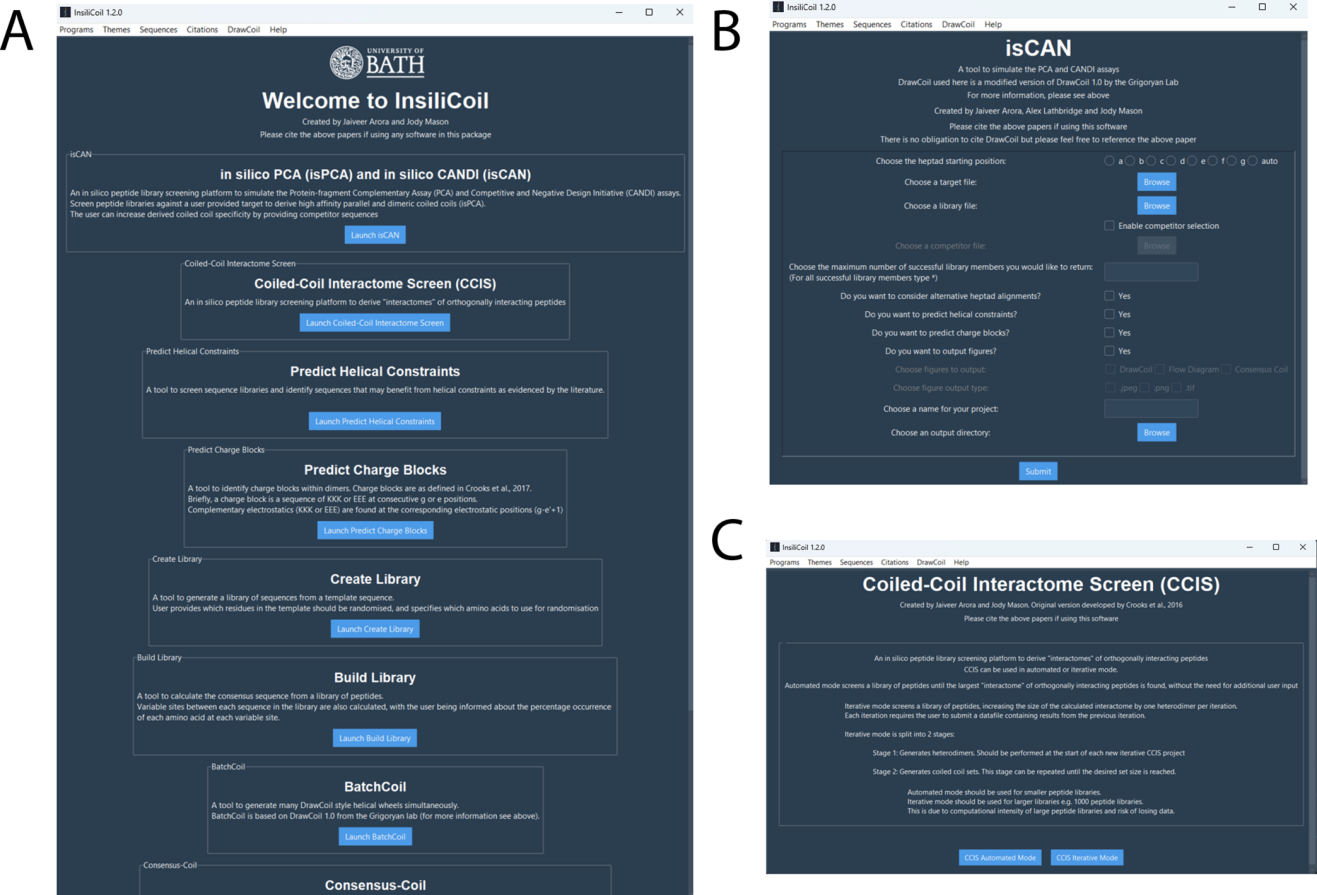
The InsiliCoil graphical user interface (GUI). (A) The InsiliCoil homepage, from which users can access all integrated modules. This includes the flagship screening tools: (B) isCAN, used to identify selective coiled coil inhibitors through simulated intracellular screening, and (C) Coiled-Coil Interactome Screen (CCIS), used to discover orthogonally interacting coiled coil networks. From here, users can choose to use either the automated or iterative CCIS pipeline. The GUI is built in Python using tkinter with styling provided by ttkbootstrap, offering a user-friendly, cross-platform interface.

### *In Silico* Competitive and Negative Design (isCAN)

The first flagship module, *isCAN*, is a high-throughput screening engine that computationally simulates intracellular CC formation. It was developed as an *in silico* approximation of the protein-fragment complementation assay (PCA),^56,57^ incorporating the Competitive and Negative Design Initiative (CANDI) approach to improve selectivity. Originally introduced by Lathbridge and Mason in 2018,^49^ isCAN was the first platform to model CC targeting peptide inhibitors with partner-specificity at scale. The tool supports two modes:

- **isPCA**: screens a peptide library against a specified target, and evaluates both on-target (TL) and off-target (TT, LL) interactions.
- **isCAN**: builds on isPCA by including competitor sequences, adding additional selectively constraints (TC and LC interactions)

In both modes, thermal stabilities (*T*_m_ values) are predicted using the bCIPA algorithm.^43,44^ Each peptide is then scored using Δ*T*_m_ values—the difference between its predicted *T*_m_ for the on-target heterodimer and its most stable off-target interaction, resulting in four Δ*T*_m_ scores per library member (Figure 2). In its original implementation, a sequence was considered a hit only if all four required Δ*T*_m_ values exceeded a user-defined threshold.^49^ However, this approach proved sensitive to cutoff selection: overtly stringent thresholds yielded no hits, while more permissive settings reduced specificity.

**Figure 2:**
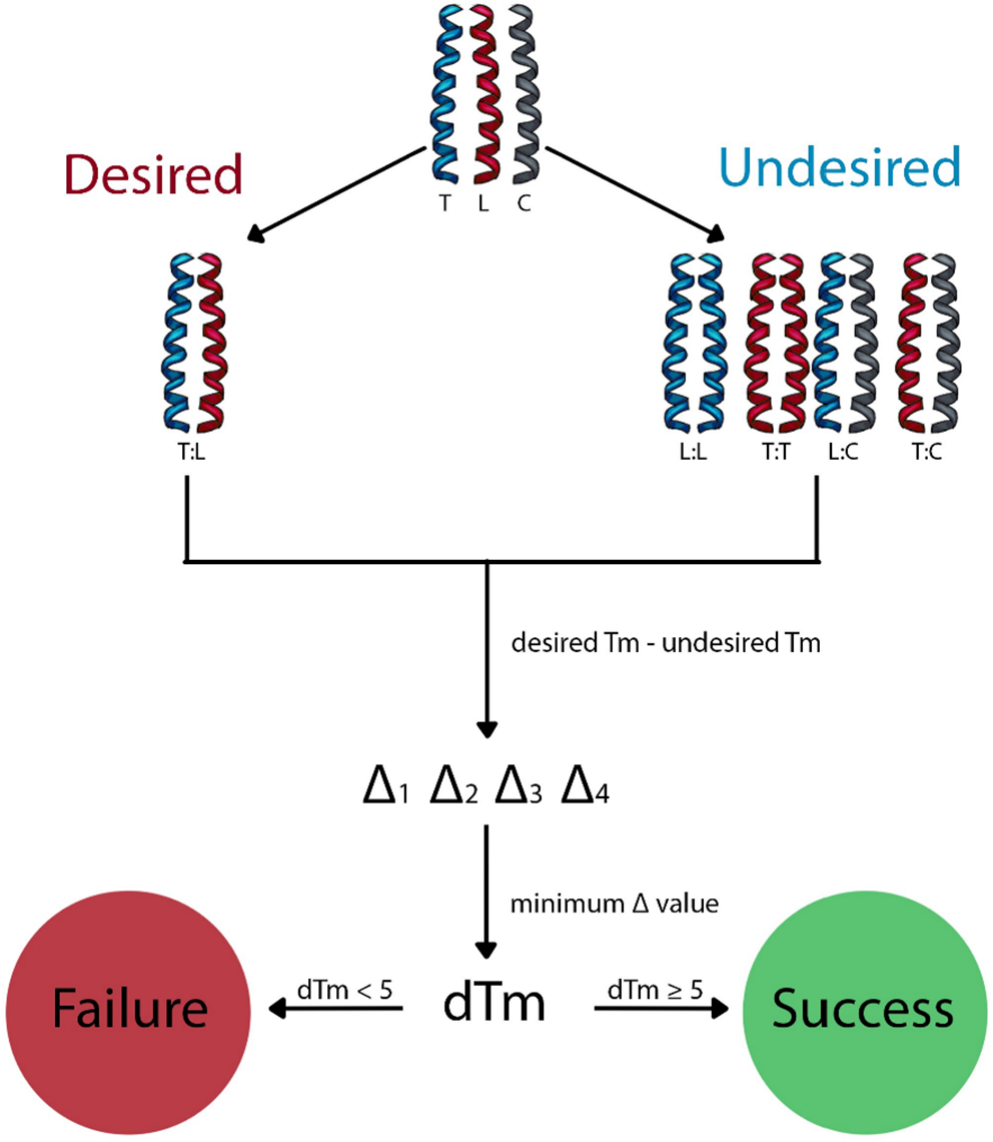
Overview of the isCAN screening protocol. Thermal stabilities (*T*_m_) are predicted using the bCIPA algorithm^41,42^ for all relevant dimeric states. The desired interaction (target–library; **T–L**) is assessed alongside multiple undesired states, including target homodimer (**T–T**), library homodimer (**L–L**), and interactions with competitors (**T–C**, **L–C**). For each library member, a Δ*T*_m_ is calculated as the difference between the on-target *T*_m_ and off-target *T*_m_. The **Δ*T*_m_** for a library member is defined as the minimum of all Δ*T*_m_ values. A peptide is considered successful if Δ*T*_m_ ≥ 5 °C. Abbreviations**:** T – target; L – library member; C – competitor.

To overcome limitations of the original implementation, we re-engineered isCAN as a streamlined, GUI-driven application (Figure 1B), eliminating the need for strict folder hierarchies and manual file paths. Improvements in memory optimization and algorithm design now allow rapid, large-scale screening: whereas the original version required 6 h to analyze 1 million peptides with four competitors, the updated isCAN processes a 60 million-member library with four competitors in under 3 h, without the need for batch partitioning or multistep protocols.

We also simplified hit selection by replacing the manually set Δ*T*_m_ threshold with a fixed cutoff of 5 °C, balancing selectivity with hit recovery. Users now specify only number of top-ranking peptides to return (e.g., the top 100 hits). Candidates are scored by Δ*T*_m_, defined as the lowest of the four Δ*T*_m_ values for a given peptide. Higher Δ*T*_m_ values reflect greater predicted specificity and stability, and sequences are ranked accordingly (Figure 2).

### New Functionalities

Several additional new features have been added to extend isCAN’s capabilities:

- **Automatic heptad frame determination** – isCAN requires all supplied peptides be register matched for accurate calculations. The automatic heptad frame determination algorithm identifies the register for each supplied peptide, and validates based on the heptad register of the target sequence. If competitors are supplied, the calculation is halted if they are not predicted to be register matched with the target sequence. Library members with predicted heptad registers differing to the target are discarded, but the isCAN calculation proceeds as expected. Manual heptad register specification remains an option, however, if manually specified, no register validation is conducted.
- **Helical constraint prediction** – using a standard literature-derived database, isCAN scans for sequence motifs previously shown to benefit from helix-stabilizing modifications in CC systems, such as lactam bridges or xylene-based bridges. If a match is found, the software flags the motif, recommends a constraint type, and links to the supporting reference. To ensure relevance, only constraints validated in CC contexts are included (currently 15 validated constraints^54,58–62^ ) with ongoing updates planned as the field evolves.
- **Charge block prediction** – isCAN now detects blocks of at least three consecutive lysine (K) or glutamate (E) residues at the ***e*** or ***g*** positions, with the corresponding complementary blocks in the target peptide at the ***g-e’+1*** positions.^8^ Though precise effects on *T*_m_ are not yet predicable, such charge block motifs have been observed to enhance dimer stability by reinforcing intermolecular attraction and intramolecular repulsion.
- **Alternate heptad alignments** – isCAN is now capable of analyzing all possible frame shifts when screening interactions. This feature is particularly useful for libraries in which the peptide is at least one heptad shorter than the target. During screening, the heptad register of the library is preserved while the peptide is systematically shifted along the target, one heptad at a time. At each iteration, the bCIPA-predicted *T*_m_ is recorded and compared across all alignments to identify the highest-scoring configuration, thereby revealing the most favorable binding arrangement (Supplementary Figure 2). When the library and target peptides are of equal length, or differ by fewer than seven residues, alternative alignment is unnecessary since it substantially increases InsiliCoil run time without affecting outcomes. For reference, enabling this feature on a 60,466,176 member benchmarking library with four competitors increased total runtime from under 3 h to approximately 18 h.

### Visual Output Enhancements

To aid interpretation, we integrated advanced visualization tools (Figure 3). A flow diagram now tracks each peptide’s progress from initial screen to final hit status. In addition, isCAN incorporates a modified version of *DrawCoil 1.0* ^63^ to generate standardized helical wheel diagrams.^32,48–50^ These are either for the highest-scoring hit (highest Δ*T*_m_) or as a *Consensus-Coil*, which aggregates all returned sequences and indicates conserved (fixed) versus variable sites (marked as “?”). Customization options include residue labeling, color schemes, and electrostatic bridge display. DrawCoil outputs require at least one successful hit; Consensus-Coil requires two or more.

**Figure 3:**
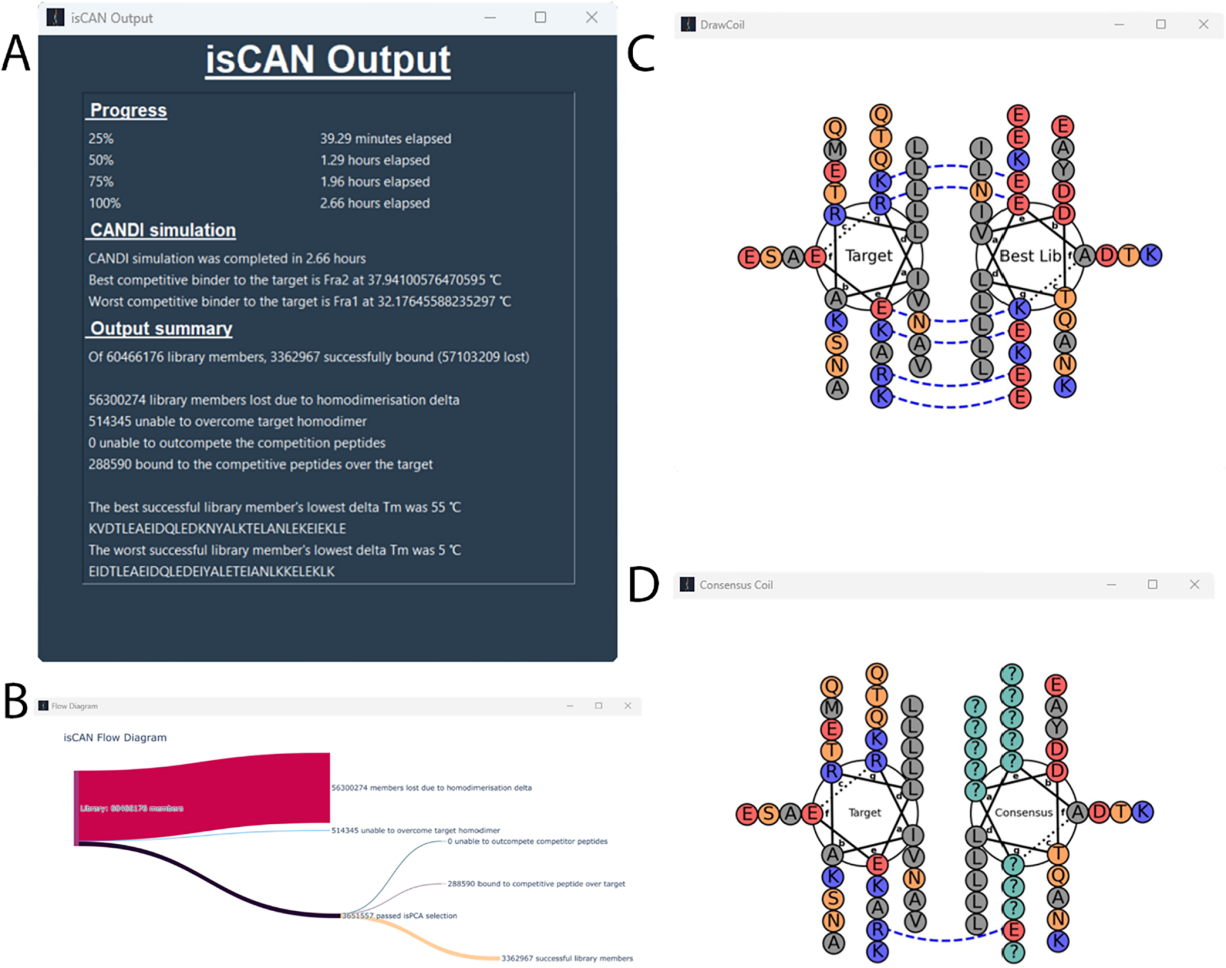
isCAN output windows. (A) Summary window displaying key results from the isCAN screen, including ranked peptide hits and associated Δ*T*_m_ values. (B) Flow diagram illustrating the distribution of library members across all binding states (T–L, L–L, T–T, L–C, T–C), showing how sequences progress through the screening pipeline. (C) DrawCoil-style helical wheel diagram^63^ depicting the interaction between the target and the best successful library member (i.e., the hit with the highest Δ*T*_m_). At least one successful member must be identified for this output to be generated. (D) Consensus-Coil diagram, also in DrawCoil style, generated from the consensus sequence of all successful library members. At least two successful hits are required for this output to be displayed. In both (C) and (D), sequence names and residue color schemes are user customizable. Abbreviations: T – target; L – library member; C – competitor.

Together, these upgrades transform isCAN into a user-friendly, robust, and scalable platform capable of screening tens of millions of peptide candidates with high selectivity, while offering rich functional and visual outputs to guide inhibitor design.

### isCAN Validation

To validate isCAN, we took two independent exercises: (1) replication of the original Lathbridge and Mason study^49^ using the redesigned software; and (2) *in silico* replication of a PCA screen conducted by Yu et al.^34^ In the first case, we recreated the 60,466,176-member peptide library from the original isCAN publication and screened it against cJun, with cFos, FosB, Fra1, Fra2 as competitors. The updated version of isCAN returned the same top ten hits as the original, confirming its computational integrity and predictive accuracy (See Supporting Information for sequences). In the second validation, we tested isCAN’s ability to reproduce the experimental workflow of Yu et al., who used PCA to discover a selective inhibitor of ATF3. Their approach exploited two distinct peptide libraries:

- An ***a***-position library (248,823 members)
- An ***e/g***-position library (59,049 members)

Together, these sampled a combinatorial space of ∼1.5 × 10^10^ sequences. To maintain parity with the published experiment, we screened the two libraries independently in *isCAN* (without competitors). The experimentally validated ATF3 inhibitor from the ***e/g*** library, ATF3W_eg, was recovered and ranked seventh by Δ*T*_m_. However, the ***a***-position library returned no successful hits. Further investigation revealed that this discrepancy stemmed from a known limitation in the bCIPA scoring algorithm: glutamine (Q), used by Yu et al. as a neutral placeholder, was penalized disproportionately in homodimers due to pairwise scoring rules. This inflated *T*_m_ values for nonspecific interactions and obscured true positives. While bCIPA does not yet account for higher-order effects (e.g., triplet interactions or charge clusters), it remains the best-validated CC scoring model to date^44,49,50,52,53^ and is retained in the current version. Alternative models are under evaluation for future updates.

To work around the scoring limitation, we generated a consensus sequence from the top 10 hits of the ***e/g*** library, identifying positional preferences (e.g., glutamate enrichment at g_1_, e_1_, g_4_, e_4_). These features were then merged with the ***a***-position library to construct a new 8,957,952-member hybrid library. Screening this refined set in isCAN successfully recovered ATF3W_aeg as the top-ranked hit (Figure 4).

**Figure 4:**
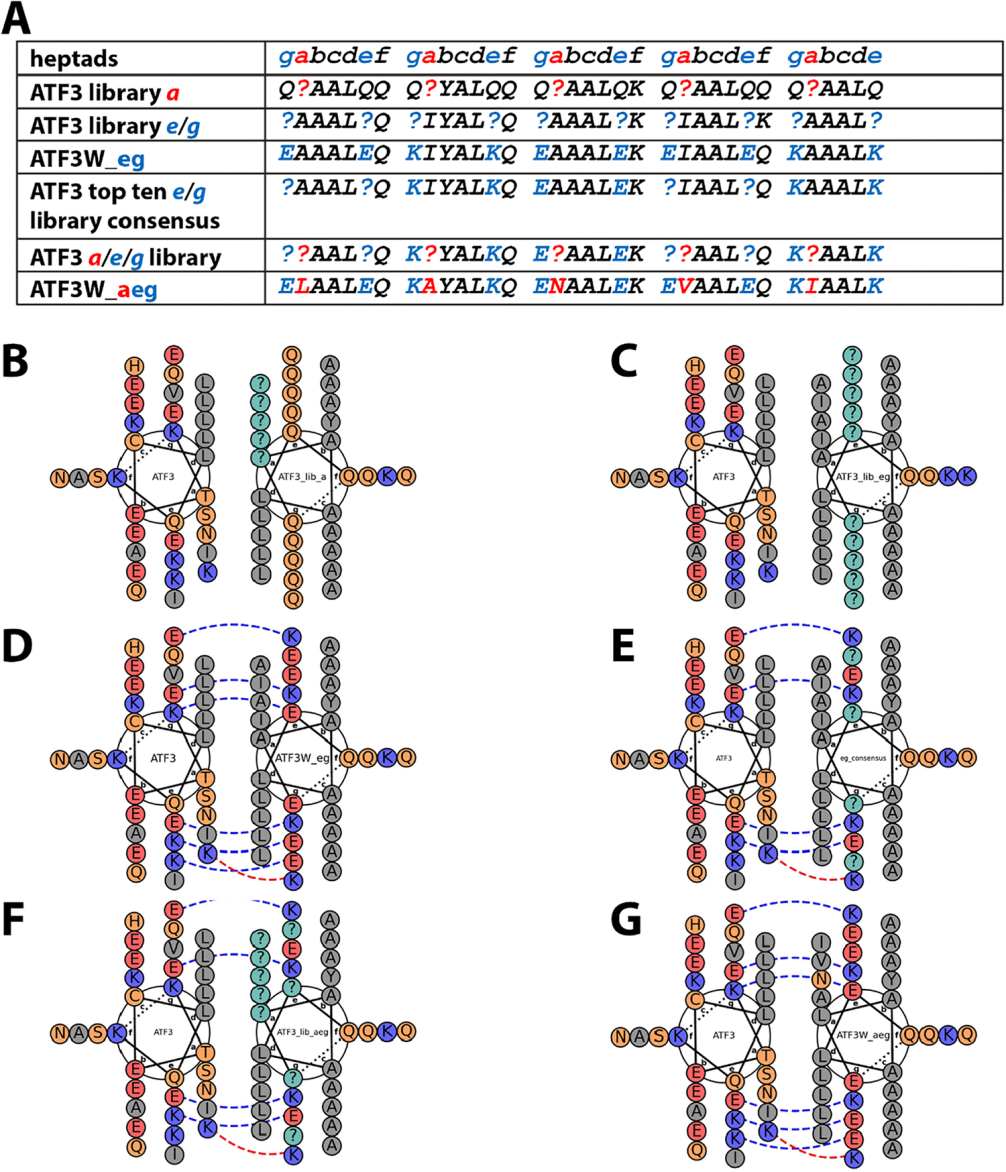
Helical wheel representations of ATF3-targeting peptide libraries and isCAN screening results. (A) Overview of library design and isCAN outcomes. The ATF3 a-position library (variable residues: FLIVYHNDSPTA) and e/g-position library (variable residues: QEK) were constructed based on the design by Yu et al.^34^ The experimentally validated peptide ATF3W_eg, recovered as the seventh-ranked hit in the isCAN screen of the ***e/g*** library, was included for benchmarking. The top ten hits from this screen were used to generate a consensus ***e/g*** sequence, which was then combined with the ***a***-position library to form the ***a/e/g*** combinatorial library (***a*** positions: FLIVYHNDSPTA; g1 and e4: QEK; e1 and g4: EQ). The top hit from this library, ATF3W_aeg, exactly matched the experimentally validated winner from Yu et al.^34^ In the sequence diagrams, ***a*** residues are highlighted in red, and ***e/g*** residues in blue. (B–G) Helical wheel diagrams corresponding to sequences described in (A). All diagrams were generated using the BatchCoil tool in InsiliCoil. Variable positions across the libraries are denoted with “?”.

Together, these results demonstrate that isCAN can recover experimentally validated coiled coil inhibitors and replicate selective binding outcomes entirely *in silico*. While limitations remain in rare edge cases, isCAN remains highly robust across diverse screening regimes. Its success in reproducing the ATF3 inhibitor highlights its predictive power and translational relevance.

As computationally assisted drug discovery (CADD) continues to transform therapeutic development,^36–38^ isCAN provides a rare example of a high-throughput motif-specific screening platform tailored to the coiled coil class long considered “undruggable”.^29,34,53^ The clinical success of peptide drugs (e.g., semaglutide, SILIQ) and CADD-designed therapies (e.g Selinexor) underscores the growing demand for such platforms. By combining scalability, specificity, and accessibility, isCAN represents a significant step forward in peptide-based PPI inhibition, particularly for transcription factors and structurally simple yet functionally complex targets.

### isCAN Alternate Heptad Alignment Validation

To validate our alternate heptad alignment algorithm, we tested a 42-member library targeting cJun, with cFos, FosB, Fra1, and Fra2 as competitors (See Supporting Information for sequences). The library comprised three sets of 14 peptides: (i) full-length 34-residue sequences matching the target and competitors, 10 of which were known isCAN hits; (ii) variants with the first heptad removed; and (iii) variants with the final heptad removed.

Of the 42 peptides, 18 were identified as successful binders. Notably, substantial differences were observed between the full length and truncated (27-residue) peptides (Table 1). The least stable successful full-length peptide exhibited *T*_m_ and Δ*T*_m_ values approximately 40 °C higher than the best performing shortened variant (*T*_m_ = 47.2 °C; Δ*T*_m_ = 8 °C), underscoring the stabilizing effect of increased heptad length and extended binding interface.

**Table 1:** isCAN Screening Results Considering Alternate Heptad Alignment Ranked by Δ*T*_m_

sequence			
gabcdef gabcdef gabcdef gabcdef gabcde	library-target *T*_m_ (°C)	library–library *T*_m_ (°C)	Δ*T*_m_ (°C)
QIDTLEA EIDQLED KNYALKT ELANLEK EIEKLE	92.1	39.3	53
QIDTLEA EIDQLED KNYALKT EIANLEK EIEKLE	91.6	38.1	52
KIDTLEA EIDQLED KNYALKT EIANLEK EIEKLE	93.8	42.7	51
KIDTLEA EIDQLED KNYALKT ELANLEK EIEKLE	94.4	43.9	51
KIDTLQA EIDQLED KNYALKT EIANLEK EIEKLE	90.7	41.2	49
QIDTLEA EIDQLED ENYALET EIANLEK EIEKLE	90.9	41.6	49
KIDTLQA EIDQLED KNYALKT ELANLEK EIEKLE	91.3	42.4	49
QIDTLEA EIDQLED ENYALET ELANLEK EIEKLE	91.5	42.8	49
KIDTLKA EIDQLED KNYALKT ELANLEK EIEKLQ	88.0	35.9	49
KIDTLEA EIDQLED KNYALKT ENANLEK EIEKLE	87.8	30.6	48
KIDTLEA EIDQLED KNYALKT EIANLE	47.2	9.9	8
KIDTLEA EIDQLED KNYALKT ELANLE	48.0	11.4	8
EIDQLED KNYALKT ELANLEK EIEKLQ	45.4	12.2	6
QIDTLEA EIDQLED KNYALKT ELANLE	45.7	6.9	6
QIDTLEA EIDQLED ENYALET ELANLE	45.5	11.2	6
QIDTLEA EIDQLED KNYALKT EIANLE	45.0	5.4	5
QIDTLEA EIDQLED ENYALET EIANLE	44.7	9.7	5
KIDTLQA EIDQLED KNYALKT ELANLE	44.6	9.5	5

The binding interfaces predicted by our alternate heptad alignment algorithm were further validated using AlphaFold 3 ^46^ for the eight shortened peptides that passed the isCAN screening. The predicted models showed high confidence, with plDDT scores generally >90, (remaining residues 70–90), and an average ipTM and pTM values of 0.67 and 0.74, respectively (lowest values 0.55 and 0.67). In all eight cases the binding interface identified by AlphaFold 3 matched the alignment of our alternative heptad alignment implementation. The full set of model confidence metrics and AlphaFold outputs are provided in the Supporting Information.

### Coiled Coil Interactome Screen

The second flagship tool within InsiliCoil is the Coiled-Coil Interactome Screen (CCIS), designed to identify large sets of orthogonally interacting CCs from user-defined libraries. CCIS builds on the computational interactome screen (CIS) introduced by Crooks et al.,^8,51^ but simplifies and accelerates the workflow for greater utility and speed.

As in CIS, CCIS calculates melting temperatures (*T*_m_ values) for all pairwise interactions within a peptide library. Homodimers are excluded, and heterodimers are filtered using two key criteria: (1) a minimum *T*_m_ threshold to remove weak binders, and (2) a selectivity cutoff (Δ*T*_m_) that limits off-target binding—similar to the filtering strategy used in isCAN (Figure 2). Optionally, users may exclude antiparallel dimers for greater specificity. Unlike CIS, which requires four additional user-defined parameters, CCIS deliberately limits inputs to these core filters to streamline usage and reduce overfitting. This design is informed by the characteristics of the bCIPA algorithm, which performs best when classifying strong versus weak interactions^43,44,49,50,52,53^ but tends to overestimate *T*_m_ values for long peptides and underestimate for short ones.^8,49,51^ By minimizing tuning parameters, CCIS ensures consistent performance across diverse libraries. To address the combinatorial complexity of large-scale pairing, CCIS employs an iterative screening strategy. After identifying valid heterodimers, pairwise combinations are screened and progressively assembled into larger sets (e.g., quadruples, octuples). At each stage, only valid combinations are retained, dramatically reducing computational burden. For example, a 1536-member library yields ∼1.2 million heterodimers. Without filtering, this generates ∼695 billion possible combinations—but if just 5% of dimers are excluded initially, this drops to ∼627 billion comparisons, saving ∼68 billion computations. Gains are even greater for larger sets.

A major advance in CCIS lies in automation and user interface design. Two operation modes are available:

- **CCIS automated**: runs a fully automated pipeline from a single input. The software iteratively screens increasing set sizes, continuing only if at least two valid sets are identified. Final outputs include all top-ranked sets at the largest valid size, a summary table, and an optional heatmap of the best interactome (Figure 5).
- **CCIS iterative**: mirrors the automated pipeline but requires user input between stages. A data.json file is generated at each step, allowing users to resume screening, adjust thresholds, or recover progress after hardware interruptions. This mode suits large libraries, lower-spec machines, or workflows where gradual relaxation of thresholds is preferred.

**Figure 5:**
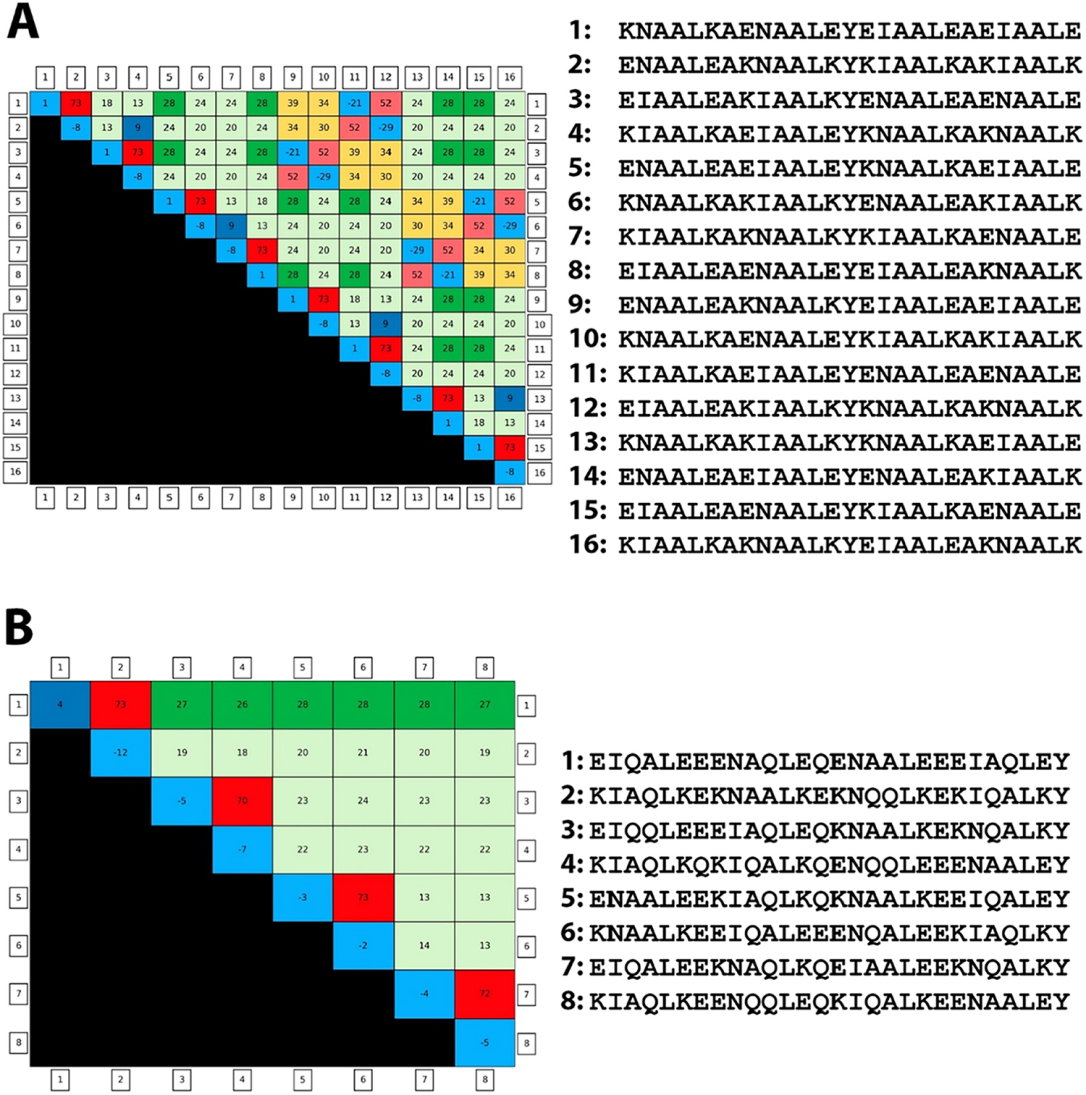
CCIS-predicted coiled coil interactomes and corresponding heatmaps. (A) Predicted interactome generated using the 16-peptide library described by Crooks et al.^8^ (B) Predicted interactome generated using the 8-peptide set described by Gradišar and Jerala.^13^ In both cases, CCIS successfully reproduces the orthogonal interactomes reported in the original experimental studies. Heatmaps display *T*_m_ values for all valid interactions. Sets were identified using CCIS automated with user-defined minimum *T*_m_ and Δ*T*_m_ thresholds (see Methods) and visualized using the integrated heatmap generation tool.

In both modes, users can generate a heatmap of top-ranked interactomes by Δ*T*_m_. In automated mode, the heatmap corresponds to the largest valid set; in iterative mode, it reflects the currently screened set size.

Additional features include charge block prediction, applied across all interactomes. Any predicted blocks are flagged and saved within the data.json to prevent redundant computation. By enhancing speed, automation, and accessibility, CCIS transforms interactome design into a scalable and user-friendly process. It is particularly valuable for researchers engineering orthogonal CC scaffolds in synthetic biology, cell targeting, or signaling applications.

### Coiled Coil Interactome Screen Validation

To validate CCIS, we applied two approaches: reproducing a published predicted interactome and reconstructing an experimentally verified one not previously predicted by CIS.

First, we recreated the octuple interactome from Crooks et al.,^8^ originally predicted using CIS. Instead of screening the full 1,536-member library, we focused on the 16 peptides that formed the final set. CCIS reproduced the identical interactome via the automated pipeline (Figure 5A). Second, we tested whether CCIS could identify an experimentally verified interactome not previously predicted computationally: the eight-peptide set from Gradišar et al.,^13^ CCIS successfully reconstructed the complete interactome (Figure 5B), demonstrating its power to identify valid CC networks beyond the original CIS scope.

As with isCAN, bCIPA’s predicted *T*_m_ values occasionally diverge from experimental data and struggle to discriminate between very similar sequences. While Crooks et al. addressed this with qCIPA,^8^ which improves within-library accuracy, it imposes strict residue constraints (e.g., only I/N at ***a***, L at ***d***, E/K at ***e/g***), limiting its generalizability. In contrast, bCIPA supports unrestricted sequence diversity and remains a top-performing classifier across diverse data sets.^13,43,44,49,50,52,53^ For this reason, it remains the default predictive model in CCIS, with future versions of InsiliCoil aiming to integrate qCIPA and others for broader user choice. This is similar to multimodel frameworks in phylogenetic software.

As the demand grows for orthogonal CCs smart materials,^64^ molecular electronics,^16^ biosensors,^13^ and therapeutic delivery,^17,19^ so does the need for robust computational tools. Although several algorithms model or predict CC interactions,^5,7,10,39,40,42–44^ few offer accessible high-throughput pipelines. CCIS fills this gap by evolving CIS into a fully automated, scalable, and experimentally aligned platform for interactome discovery. Despite minor limitations in *T*_m_ calibration, CCIS delivers state-of-the-art classification and broad utility across applications ranging from protein logic gates and diagnostics to smart delivery systems and synthetic cell circuits.

### Create Library

The *Create Library* tool enables rapid and flexible design of peptide libraries through user-defined sequence variability. Users input a backbone sequence and designate specific heptad positions (***a*** *–* ***g***) to vary, along with permitted amino acid options at each position. This allows for both focused and broad combinatorial library designs tailored to structural or functional hypotheses. For example, users may vary core ***a/d*** positions to modulate dimer stability, or ***e/g*** positions to tune electrostatic specificity. The tool then generates all permutations of the specified design space and exports the resulting library in FASTA format, fully compatible with downstream isCAN and CCIS screening. This functionality supports a range of strategies, including alanine or glutamine scans, mimicking experimental combinatorial libraries, or integrating data from structural models or machine learning predictions. By automating the generation of highly customizable sequence libraries, *Create Library* accelerates the design–build–test cycle and improves reproducibility in coiled coil research.

### Build Library

*Build Library* operates as the inverse of *Create Library*. Rather than generating sequences, it analyses an existing library such as hits from isCAN or naturally occurring CC sequences to extract consensus motifs, positional variability, and residue enrichment or depletion. The tool outputs a consensus sequence and position-specific frequency matrix, highlighting conserved residues likely important for binding, and variable sites suitable for further optimization. This supports second-generation library design, sequence–function analysis, and rational refinement based on prior screening results. *Build Library* also integrates with *Consensus-Coil* for visual representation of residue trends, but can be run independently as well. When used with *Consensus-Coil*, it automatically provides background calculations for generating sequence-based helical wheel projections.

### BatchCoil

*BatchCoil* extends the functionality of *DrawCoil* to support high-throughput generation of helical wheel diagrams from bulk FASTA input. It processes multiple peptide sequences simultaneously, generating visual projections that display residue positions and electrostatic interactions across a library. This is particularly useful for inspecting isCAN outputs, comparing designed motifs, or creating publication-quality figures. Users retain full control over graphical parameters including residue labels, color schemes, and bridge displays, consistent with the *DrawCoil* interface. *BatchCoil* eliminates the need for manual entry, enabling efficient visual screening of dozens to thousands of sequences. It is especially valuable for identifying shared motifs, highlighting inconsistencies, and validating structure-based design hypotheses across large data sets.

### Automatic Heptad Frame Determination

Details underlying the automatic heptad frame determination algorithm and validation are discussed in the Supporting Information. For all modules other than isCAN (discussed above), automatic heptad frame determination requires that all input peptides share the same heptad register. If any peptide is predicted to occupy a different register, the calculation is halted and an invalid_sequences.csv file is written to the specified output directory. The algorithm identifies the register containing the largest number of peptides as the valid register, with all remaining peptides classified as invalid. The output file lists each invalid peptide and its predicted register, enabling users to adjust or curate their dataset prior to re-running the analysis.

### Heptad Scanning (isCAN only)

Evaluates all permissible heptad alignments between each library peptide and the target sequence. For each alignment, stability metrics are calculated and the Δ*T*_m_ between LT and TT or LL (or additionally LC and TC if supplied) is determined. The alignment yielding the most favourable (i.e., highest) Δ*T*_m_ is retained for downstream scoring. This allows the software to account for alternative registers that may produce more optimal coiled coil interactions than the default alignment.

### bCIPA Limitations

The bCIPA algorithm forms the core of the screening modules within InsiliCoil, yet it exhibits a few limitations. Although it reliably discriminates between interacting and noninteracting CCs,^43,44,50^ its accuracy in predicting *T*_m_ values decreases under conditions that diverge from those used during training. This is namely ***a*** register, 32-residue peptides at a total peptide concentration of 150 μM and pH 7.0. For instance, dimers 5–6 and 7–8 from Figure 5B have ***a*** bCIPA predicted *T*_m_ values of 73 and 72 °C, respectively, yet experimentally determined *T*_m_ values of approximately 40 °C under different conditions (25 μM of each peptide, pH 7.0, ***g*** register four-heptad design with an N-terminal Ser-Pro-Glu-Asp (SPED) extension and a *C*-terminal Gly).^13^ Consequently, the model performs best for CCs of four to five heptads, with predictive accuracy diminishing as peptide length substantially deviates from the training set.

These discrepancies in *T*_m_ likely stem from bCIPA’s pairwise-scoring approach, which neglects higher-order interactions. Specifically, bCIPA employs the helical propensity scale of Williams et al.,^65^ which omits intramolecular electrostatics and salt bridge effects known to strongly influence dimer stability.^66^ Refinement of the algorithm, could therefore involve adopting more comprehensive models of helical propensity such as the Pace and Scholtz model,^67^ which integrates multiple experimental data sets, including AGADIR,^68,69^ to capture electrostatic contributions.

Further improvements could also arise from retraining bCIPA on a broader data set, encompassing peptides of varied lengths and compositions to optimize the weighting scheme. Moreover, incorporating complementary algorithms such as qCIPA^8^ or iCIPA,^70^ would expand flexibility within InsiliCoil, enabling selection of the most appropriate predictor for a given system, akin to multimodel frameworks used in phylogenetic analysis. However, both qCIPA and iCIPA currently impose restrictions on residue selection within designed CCs.

Despite these limitations, bCIPA-powered InsiliCoil modules consistently reproduce experimental outcomes across all parameters except *T*_m_ prediction. Accordingly, due to its broad applicability and proven discriminatory power, bCIPA remains the default predictive model implemented within InsiliCoil.

## Conclusions

Despite growing recognition of coiled coil (CC) proteins as versatile building blocks in both therapeutic discovery and synthetic biology, the field has long lacked a unified, user-friendly, and high-throughput computational platform for their systematic design, screening, and visualization. InsiliCoil directly addresses this gap by providing not only a practical toolkit, but a broadly applicable framework for controlling helix–helix interactions in both natural and engineered systems.

At its core are two flagship modules: isCAN, a robust platform for the rational design of peptide-based CC inhibitors, and CCIS, a scalable screening tool for discovering orthogonally interacting CC networks. These are supported by a suite of complementary utilities, including Create Library, Build Library, Predict Helical Constraints, and BatchCoil, among others. Together these enable a complete design–screen–analyze workflow. This integration ensures broad relevance, spanning therapeutic peptide discovery through to synthetic circuit construction and modular biomaterial design.

By combining predictive accuracy, scalability, and accessibility, InsiliCoil transforms the way helix-mediated PPIs can be interrogated and engineered. Integration into existing synthetic biology pipelines will accelerate identification of inhibitors against traditionally “undruggable” transcription factors, while also supporting the modular assembly of logic systems, delivery platforms, and programmable scaffolds. In this sense, InsiliCoil plays for coiled coils a role analogous to AlphaFold for structural prediction or MOE for ligand design: it provides a unifying, widely accessible foundation that will catalyze innovation across synthetic biology, biotechnology, and medicine.

## Methods

### InsiliCoil Development

InsiliCoil was developed in Python 3.12.5 and features a fully integrated graphical user interface (GUI) built using the built-in tkinter library, with styling enhancements implemented via ttkbootstrap v1.10.1.^71^ The software is modular in design, allowing for ease of maintenance and extensibility.

All numerical operations within InsiliCoil are powered by NumPy v2.1.0,^72^ ensuring efficient array manipulation and computation across all screening and visualization modules. The interactive flow diagrams produced in the *isCAN* module are generated using Plotly v6.0.0 ^73^ and exported using Kaleido v0.2.1.^74^ All other visual outputs, including helical wheel diagrams, summary plots, and structure-based illustrations, are produced using Matplotlib v3.10.0.^75^

The DrawCoil, BatchCoil, and Consensus-Coil modules are based on a Python-converted version of the original *DrawCoil 1.0* script,^63^ extensively modified for batch processing, integration, and user customization within the InsiliCoil framework.

The CCIS heatmap visualization is created using Seaborn v0.13.2,^76^ which leverages a Matplotlib backend. Data is structured and supplied to this visualization engine using pandas v2.2.3.^77^ Image handling within the GUI—including generation, preview, and export of visual outputs—is managed using the Pillow v11.0.0 ^78^ image processing library.

To ensure platform accessibility, the software was compiled into a standalone Windows executable (.exe) using Nuitka v2.6,^79^ and into a .app bundle for macOS using PyInstaller v6.11.1.^80^ For Windows distribution, an installer was packaged using Inno Setup v6.4.0.^81^

### Speed Testing

All speed benchmarking was performed using the Windows version of InsiliCoil on a Lenovo Legion 5i laptop under controlled conditions. The hardware specifications were as follows:

- CPU: Intel Core i5–10300H (quad-core, 2.5 GHz base clock)
- Memory: 2 × 8 GB DDR4 SODIMM RAM (2933 MT/s)
- Storage: 500 GB NVMe SSD
- GPU: NVIDIA GeForce GTX 1650

To ensure consistency, all speed tests were conducted with no other applications running in the background. The laptop remained connected to mains power throughout the tests and was configured to operate in high performance mode. Timing measurements were obtained using InsiliCoil’s built-in job timer, which records the total time required to complete all calculations for a given screen. These timings were independently verified using a manual stopwatch. All results reported represent the average of three independent runs to account for minor variability in performance.

### isCAN Validation Experiments

All peptide libraries used for isCAN validation were generated using the *Create Library* tool within InsiliCoil.^8,49,51,53^ The cFos-based library was constructed following the design described in Lathbridge and Mason.^49^ Briefly, a cFos-derived template sequence was used, incorporating variability at the ***a*** positions with the amino acids I, L, V, and N, and at the ***e/g*** positions with Q, E, and K. The full template sequence is provided in the Supporting Information.

Similarly, ATF3 ***a*** and ATF3 ***e/g*** libraries were generated based on the design by Yu et al.^34^ The ATF3 a library employed variability at ***a*** positions with the residues F, L, I, V, Y, H, N, D, S, P, T, and A, while the ***e/g*** library used Q, E, and K at all ***e*** and ***g*** positions. Template sequences for both libraries are provided in Figure 4.

The cFos-based library was screened against a cJun target in the presence of four competitors: cFos, FosB, Fra1, and Fra2. Both ATF3 libraries were screened against an ATF3 target, without competitors. All target and competitor sequences are available in the Supporting Information. isCAN was configured to return the top 100 successful hits, with alternate heptad alignment, helical constraint and charge block prediction disabled. Results from the cFos-based library were compared to the top ten hits reported in.^49^ Results from the ATF3 libraries were compared to PCA-validated hits described in.^34^

To generate a refined library for enhanced screening, the top ten hits from the ATF3 ***e/g*** library were extracted and analyzed using the Consensus-Coil tool within InsiliCoil to determine a consensus sequence (Figure 4). The resulting consensus sequence was combined with the ATF3 ***a*** library to generate an ATF3 ***a/e/g*** combinatorial library. In this library, ***a*** positions used FLIVYHNDSPTA, ***g1*** and ***e4*** positions used QEK, and ***e1*** and ***g4*** positions used QE. This combined library was screened using isCAN against ATF3, with settings identical to the previous runs and without competitors. The resulting hits were compared to the validated PCA outcomes from.^34^

### isCAN Alternate Heptad Alignment Validation Experiments

The library used was generated from previously validated 34-residue sequences, ten of which were known to bind to cJun and four known to fail during screening. Two additional sets of 14 peptides were derived from this initial collection: one with the first heptad removed, and the other with the final heptad removed. The three resulting 14-member sets were combined into a 42-member library, which was screened against a cJun target in the presence of four competitors: cFos, FosB, Fra1, and Fra2. All peptide sequences are provided in the Supporting Information.

isCAN was configured to return all successful hits with the alternate heptad-alignment option enabled, and both helical-constraint and charge-block prediction features disabled. Results for successful full length (34-residue) peptides were compared to those obtained with alternate heptad alignment disabled.

The binding interfaces of the shortened (27-residues) successful peptides were subsequently verified using AlphaFold 3,^46^ accessed via the AlphaFold Server (https://alphafoldserver.com/). Models were generated using the default random seed, and visualized using the Server’s integrated model viewer. Detailed output statistics are available in the Supporting Information.

### bCIPA Component Analysis

To investigate the individual contributions of each component of the bCIPA algorithm—namely helical propensity (HP), core hydrophobicity (C), and electrostatic interactions (ES)—a standalone Python script was created based on the original implementation of bCIPA.^43,44,49,65,82–84^ The final melting temperature (*T*_m_) is typically calculated using the following equation, adjusted to Celsius

2 $$T_{m}=a\times \mathrm{HP}+b\times C+c\times \mathrm{ES}+d-273.15$$

where ***a*** = 81.3256, ***b*** = −10.5716, ***c*** = −4.7771, and ***d*** = −29.1320.

For this analysis, we modified the bCIPA code to bypass the final *T*_m_ calculation and instead return a Python dictionary containing the unweighted and weighted values for each component:
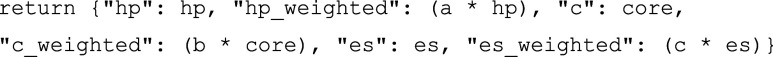


This allowed for direct comparison of the relative contribution of each component to the overall *T*_m_ prediction for a given dimer. These values were then analyzed to identify dominant terms responsible for deviations between predicted and experimentally observed *T*_m_s, particularly in isCAN and CCIS validation screens.

### CCIS Validation Experiments

To validate the performance of CCIS, two curated libraries were used. The first was the 16-peptide library from Crooks et al.,^8^ which yields an orthogonal octuple. The second was the 8-peptide library from Gradišar and Jerala,^13^ which defines a validated quadruple of orthogonally interacting CCs. All peptide sequences are provided in the Supporting Information.

Each library was screened using CCIS automated, with charge block predication disabled. Heatmap generation enabled to visualize interactomes. Screening parameters were selected based on the characteristics of each system:

- For the 16-peptide library: Minimum desired *T*_m_: 30 °C, Δ*T*_m_ (differential cutoff): 20 °C
- For the 8-peptide library: Minimum desired *T*_m_: 70 °C, Δ*T*_m_ (differential cutoff): 40 °C

The heatmaps and interactome structures returned by CCIS were then compared to the reference interactomes described in their respective publications. These experiments confirmed that CCIS could accurately reproduce known orthogonal sets, validating its ability to identify noncross-reactive CC networks under experimentally relevant conditions.

## Supplementary Material



## Data Availability

InsiliCoil is freely available for academic and noncommercial use. Windows (.exe) and macOS (.app) versions, along with full documentation, installation instructions, and example data sets, can be downloaded from https://people.bath.ac.uk/jm2219/biology/InsiliCoil/index.htm. The help guide can be accessed from either the Web site or through the help menu directly within InsiliCoil.

## Notes

A detailed description of the bHLHZIP leucine zipper sequence alignments and peptide sequences utilized in this work (PDF)
