# Supplementary material for: InsiliCoil: An Integrated Software Suite for Coiled Coil Design, Prediction, and Therapeutic Engineering
Source: ACS Synth Biol. 2025 Dec 10;15(2):586–98. doi: 10.1021/acssynbio.5c00678 (PMC12930490; doi:10.1021/acssynbio.5c00678)
Supplement: Supplementary file 1 [file sb5c00678_si_001.pdf]

Supporting Information for Publication

InsiliCoil: An Integrated Software Suite for Coiled Coil Design, Prediction, and Therapeutic Engineering.

Jaiveer Arora,[1] and Jody M Mason[1,2]

[1]Department of Life Sciences, University of Bath, Bath BA2 7AY, United Kingdom

[2]Address correspondence to JMM (j.mason@bath.ac.uk)

Keywords: coiled coil, protein-protein interactions, synthetic biology, computational design, orthogonal interactomes, bZIP, high-throughput *in silico* screening

bHLHZIP Leucine Zipper Sequences

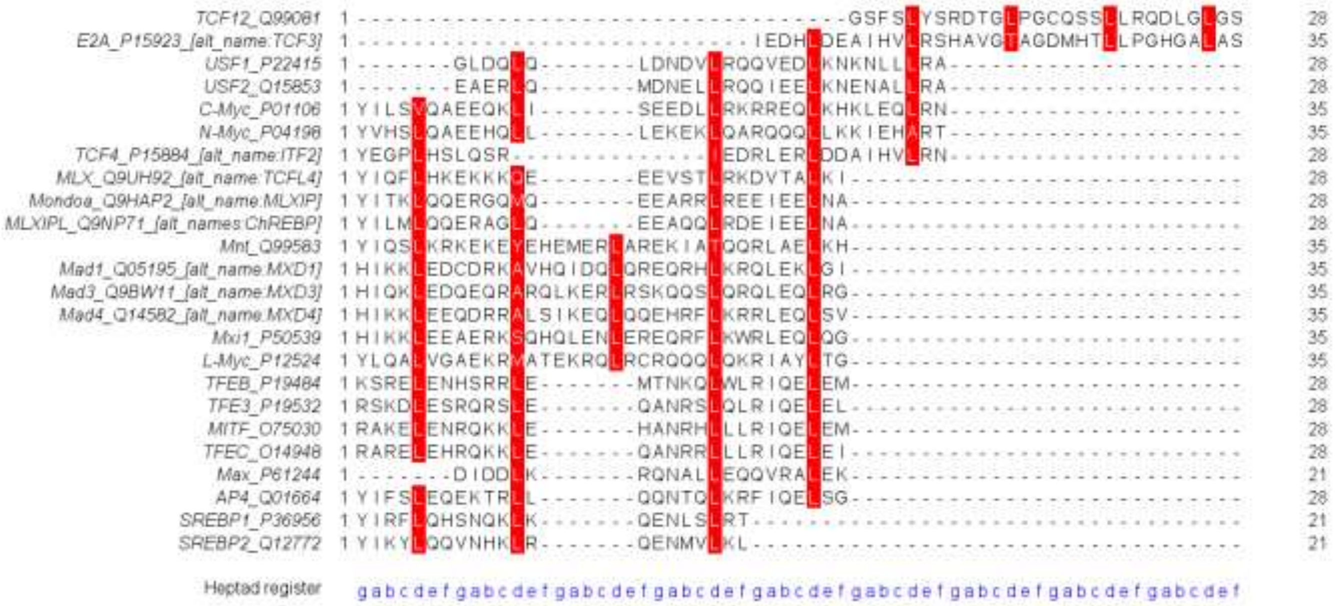

Supporting Figure 1 – bHLHZIP leucine zipper sequence alignment. *bHLHZIP protein names and UniProt codes are listed on the left in the format protein-name_UniProt-code. For proteins with alternate names included, such as MLX, the format is protein-name_UniProt-code_(alt_name: alternate-protein-name). The right margin indicates sequence length. All* ***d*** *residues are highlighted in red. Sequences were aligned using Clustal Omega 1.2.4 on 15th April 2025 accessed via EMBL-EBI Job Dispatcher (1). Settings from Clustal Omega were left as default. Sequences used in the alignment are in Supporting Table 1.*

Supporting Table 1 – bHLHZIP leucine zipper sequences.

bHLHZIP	Leucine Zipper Sequence<br>gabcdef gabcdef gabcdef gabcdef gabcdef	UniProt Code
TCF12	GSFSLYS RDTGLPG CQSSLLR QDLGLGS	Q99081
E2A (TCF3)	IEDHLDE AIHVLRS HAVGTAG DMHTLLP GHGALAS	P15923
USF1	GLDQLQL DNDVLRQ QVEDLKN KNLLLRA	P22415
USF2	EAERLQM DNELLRQ QIEELKN ENALLRA	Q15853
C-Myc	YILSVQA EEQKLIS EEDLLRK RREQLKH KLEQLRN	P01106
N-Myc	YVHSLQA EEHQLLL EKEKLQA RQQQLLK KIEHART	P04198
TCF4 (ITF2)	YEGPLHS LQSRIED RLERLDD AIHVLRN	P15884
MLX (TCFL4)	YIQFLHK EKKKQEE EVSTLRK DVTALKI	Q9UH92
Mondoa (MLXIP)	YITKLQQ ERGQMQE EARRLRE EIEELNA	Q9HAP2
MLXIPL (ChREBP)	YILMLQQ ERAGLQE EAQQLRD EIEELNA	Q9NP71
Mnt	YIQSLKR KEKEYEH EMERLAR EKIATQQ RLAELKH	Q99583
Mad1 (MXD1)	HIKKLED CDRKAVH QIDQLQR EQRHLKR QLEKLGI	Q05195
Mad3 (MXD3)	HIQKLED QEQRARQ LKERLRS KQQSLQR QLEQLRG	Q9BW11
Mad4 (MXD4)	HIKKLEE QDRRALS IKEQLQQ EHRFLKR RLEQLSV	Q14582
Mxi1	HIKKLEE AERKSQH QLENLER EQRFLKW RLEQLQG	P50539
L-Myc	YLQALVG AEKRMAT EKRQLRC RQQQLQK RIAYLTG	P12524
TFEB	KSRELEN HSRRLEM TNKQLWL RIQELEM	P19484
TFE3	RSKDLES RQRSLEQ ANRSLQL RIQELEL	P19532
MITF	RAKELEN RQKKLEH ANRHLLL RIQELEM	O75030
TFEC	RARELEH RQKKLEQ ANRRLLL RIQELEI	O14948
Max	DIDDLKR QNALLEQ QVRALEK	P61244
AP4	YIFSLEQ EKTRLLQ QNTQLKR FIQELSG	Q01664
SREBP1	YIRFLQH SNQKLKQ ENLSLRT	P36956
SREBP2	YIKYLQQ VNHKLRQ ENMVLKL	Q12772

Peptide sequences

Supporting Table 2 – Target and competitor sequences used for isCAN validation.

**Heptads**	gabcdef gabcdef gabcdef gabcdef gabcde
**cJun**	RIARLEE KVKTLKA QNSELAS TANMLRE QVAQLK
**cFos**	LTDTLQA ETDQLED EKYALQT EIANLLK EKEKLE
**FosB**	LTDRLQA ETDQLEE EKYELES EIAELQK EKERLE
**Fra1**	LTDFLQA ETDKLED EKYGLQR EIEELQK QKERLE
**Fra2**	LTEKLQA ETEELEE EKYGLQK EIAELQK EKEKLE
**ATF3**	KTECLQK ESEKLES VNAELKA QIEELKN EKQHLI

Supporting Table 3 – cFos-based library used to validate isCAN. *This library is from (2). The total library size is 60,466,176 sequences.*

**Heptads**	gabcdef gabcdef gabcdef gabcdef gabcde
**Template sequence**	??DTL?A ??DQL?D ??YAL?T ??ANL?K ??EKL?
***a* residue options**	ILVN
***e* residue options**	QEK
***g* residue options**	QEK

Supporting Table 4 – Top ten hits from a cFos-based library targeting cJun. *The hits are from (2). The same hits were identified in our top ten hits when validating isCAN using the same library targeting cJun. The design for the library can be found in Supporting Table 3.*

**Hit Rank**	**Peptide**<br>gabcdef gabcdef gabcdef gabcdef gabcde
**1**	QIDTLEA EIDQLED KNYALKT ELANLEK EIEKLE
**2**	KIDTLEA EIDQLED KNYALKT EIANLEK EIEKLE
**3**	QIDTLEA EIDQLED KNYALKT EIANLEK EIEKLE
**4**	KIDTLEA EIDQLED KNYALKT ELANLEK EIEKLE
**5**	KIDTLQA EIDQLED KNYALKT EIANLEK EIEKLE
**6**	QIDTLEA EIDQLED ENYALET EIANLEK EIEKLE
**7**	KIDTLQA EIDQLED KNYALKT ELANLEK EIEKLE

**8**	QIDTLEA EIDQLED ENYALET ELANLEK EIEKLE
**9**	KIDTLEA EIDQLED KNYALKT ENANLEK EIEKLE
**10**	KIDTLKA EIDQLED KNYALKT ELANLEK EIEKLQ

Supporting Table 5 – ATF3 **a** ***library used for isCAN validation.*** *Library is from (3). The total library size is 248,832 sequences.*

**Heptads**	gabcdef gabcdef gabcdef gabcdef gabcde
**Template sequence**	Q?AALQQ Q?YALQQ Q?AALQK Q>AALQQ Q?AALQ
a **residue options**	FLIVYHNDSPTA

Supporting Table 6 – ATF3 **e/g** ***library used for isCAN validation.*** *Library is from (3). The total library size is 59,049 sequences.*

**Heptads**	gabcdef gabcdef gabcdef gabcdef gabcde
**Template sequence**	?AAAL?Q ?IYAL?Q ?AAAL?K ?IAAL?K ?AAAL?
**e residue options**	QEK
g **residue options**	QEK

Supporting Table 7 – Consensus sequence for the top ten isCAN hits from the ATF3 **e/g** ***library against an ATF3 target.*** *The design for the ATF3* e/g *library is from (3) and can be found in Supporting Table 6 . Residues* ***g1*** *and* ***e4*** *are highlighted in red. Residues* ***e1*** *and* ***g4*** *are highlighted in blue.*

**Heptads**	gabcdef gabcdef gabcdef gabcdef gabcde
**Consensus sequence**	?AAAL?Q KIYALKQ EAAALEK ?IAAL?Q KAAALK
***g*1 residue options**	QEK
e1 **residue options**	QE
g4 **residue options**	QE
e4 residue options	QEK

Supporting Table 8 – ATF3* a/e/g *library used for isCAN validation. *The library was constructed by combining the ATF3* a *library (Supporting Table 5) with the consensus sequence of the top ten ATF3* ***e/g*** *library hits (Supporting Table 7). Residues* ***g1*** *and* ***e4*** *are highlighted in red. Residues* ***e1*** *and* ***g4*** *are highlighted in blue.*

**Heptads**	gabcdef gabcdef gabcdef gabcdef gabcde
**Template sequence**	??AAL?Q K?YALKQ E?AALEK ??AAL?Q K?AALK
***a* residue options**	FLIVYHNDSPTA
***g*1 residue options**	QEK
***e1* residue options**	QE
***g4* residue options**	QE
e4 residue options	QEK

Supporting Table 9 – Experimental hits used for isCAN validation. *All hits are from (3). ATF3W_a hit was derived from protein-complement fragmentation assay (PCA) for the ATF3* ***a*** *library (Supporting Table 5) against an ATF3 target. ATF3W_eg hit was derived from PCA for the ATF3* ***e/g*** *library (Supporting Table 6) against an ATF3 target. ATF3W_aeg was produced by combining ATF3W_a and ATF3W_eg (3).* ***B***, ***c***, ***d*** *and* ***f*** *residues were kept the same.* ***A*** *residues were inherited from ATF3W_a and* ***e/g*** *residues were inherited from ATF3W_eg.*

**Heptads**	gabcdef gabcdef gabcdef gabcdef gabcde
**ATF3W_a**	QLAALQQ QAYALQQ QNAALQK QVAALQQ QIAALQ
**ATF3W_eg**	EAAALEQ KIYALKQ EAAALEK EIAALEQ KAAALK
**ATF3W_aeg**	ELAALEQ KAYALKQ ENAALEK EVAALEQ KIAALK

Supporting Table 10 – Coiled coil octuple used for CCIS validation. *Peptides used are from (4).*

**Peptides**	**Sequence**<br>gabcdef gabcdef gabcdef gabcde
**1**	KNAALKA ENAALEY EIAALEA EIAALE
**2**	ENAALEA KNAALKY KIAALKA KIAALK
**3**	EIAALEA KIAALKY ENAALEA ENAALE
**4**	KIAALKA EIAALEY KNAALKA KNAALK
**5**	ENAALEA EIAALEY KNAALKA EIAALE
**6**	KNAALKA KIAALKY ENAALEA KIAALK

**7**	KIAALKA KNAALKY KIAALKA ENAALE
**8**	EIAALEA ENAALEY EIAALEA KNAALK
**9**	ENAALEA KNAALKY EIAALEA EIAALE
**10**	KNAALKA ENAALEY KIAALKA KIAALK
**11**	KIAALKA EIAALEY ENAALEA ENAALE
**12**	EIAALEA KIAALKY KNAALKA KNAALK
**13**	KNAALKA KIAALKY KNAALKA EIAALE
**14**	ENAALEA EIAALEY ENAALEA KIAALK
**15**	EIAALEA ENAALEY KIAALKA ENAALE
**16**	KIAALKA KNAALKY EIAALEA KNAALK

Supporting Table 11 – Coiled coil quadruple used for CCIS validation. *Peptides used are from (5).*

**Peptides**	**Sequence**<br>gabcdef gabcdef gabcdef gabcdef
**1**	EIQALEE ENAQLEQ ENAALEE EIAQLEY
**2**	KIAQLKE KNAALKE KNQQLKE KIQALKY
**3**	EIQQLEE EIAQLEQ KNAALKE KNQALKY
**4**	KIAQLKQ KIQALKQ ENQQLEE ENAALEY
**5**	ENAALEE KIAQLKQ KNAALKE EIQALEY
**6**	KNAALKE EIQALEE ENQALEE KIAQLKY
**7**	EIQALEE KNAQLKQ EIAALEE KNQALKY
**8**	KIAQLKE ENQQLEQ KIQALKE ENAALEY

Supporting Table 12 – Peptide Library used to validate isCAN Alternate Heptad Alignment

**Peptides**	**Sequence**<br>gabcdef gabcdef gabcdef gabcdef gabcde
**1**	QIDTLEA EIDQLED KNYALKT ELANLEK EIEKLE
**2**	KIDTLEA EIDQLED KNYALKT EIANLEK EIEKLE
**3**	QIDTLEA EIDQLED KNYALKT EIANLEK EIEKLE
**4**	KIDTLEA EIDQLED KNYALKT ELANLEK EIEKLE

**5**	KIDTLQA EIDQLED KNYALKT EIANLEK EIEKLE
**6**	QIDTLEA EIDQLED ENYALET EIANLEK EIEKLE
**7**	KIDTLQA EIDQLED KNYALKT ELANLEK EIEKLE
**8**	QIDTLEA EIDQLED ENYALET ELANLEK EIEKLE
**9**	KIDTLEA EIDQLED KNYALKT ENANLEK EIEKLE
**10**	KIDTLKA EIDQLED KNYALKT ELANLEK EIEKLQ
**11**	QIDTLQA QIDQLQD QNYALQT QLANLQK QIEKLQ
**12**	KIDTLQA QIDQLQD QNYALQT QIANLQK QIEKLQ
**13**	QIDTLQA QIDQLQD QNYALQT QIANLQK QIEKLQ
**14**	KIDTLQA QIDQLQD QNYALQT QLANLQK QIEKLQ
**15**	EIDQLED KNYALKT ELANLEK EIEKLE
**16**	EIDQLED KNYALKT EIANLEK EIEKLE
**17**	EIDQLED KNYALKT EIANLEK EIEKLE
**18**	EIDQLED KNYALKT ELANLEK EIEKLE
**19**	EIDQLED KNYALKT EIANLEK EIEKLE
**20**	EIDQLED ENYALET EIANLEK EIEKLE
**21**	EIDQLED KNYALKT ELANLEK EIEKLE
**22**	EIDQLED ENYALET ELANLEK EIEKLE
**23**	EIDQLED KNYALKT ENANLEK EIEKLE
**24**	EIDQLED KNYALKT ELANLEK EIEKLQ
**25**	QIDQLQD QNYALQT QLANLQK QIEKLQ
**26**	QIDQLQD QNYALQT QIANLQK QIEKLQ
**27**	QIDQLQD QNYALQT QIANLQK QIEKLQ
**28**	QIDQLQD QNYALQT QLANLQK QIEKLQ
**29**	QIDTLEA EIDQLED KNYALKT ELANLE
**30**	KIDTLEA EIDQLED KNYALKT EIANLE
**31**	QIDTLEA EIDQLED KNYALKT EIANLE
**32**	KIDTLEA EIDQLED KNYALKT ELANLE
**33**	KIDTLQA EIDQLED KNYALKT EIANLE
**34**	QIDTLEA EIDQLED ENYALET EIANLE

**35**	KIDTLQA EIDQLED KNYALKT ELANLE
**36**	QIDTLEA EIDQLED ENYALET ELANLE
**37**	KIDTLEA EIDQLED KNYALKT ENANLE
**38**	KIDTLKA EIDQLED KNYALKT ELANLE
**39**	QIDTLQA QIDQLQD QNYALQT QLANLQ
**40**	KIDTLQA QIDQLQD QNYALQT QIANLQ
**41**	QIDTLQA QIDQLQD QNYALQT QIANLQ
**42**	KIDTLQA QIDQLQD QNYALQT QLANLQ

Supporting Table 13 – isCAN hits from a library targeting cJUN*. Library peptides are from Supporting Table 12. Hits are ranked by dTm from highest to lowest.*

**Hit Rank**	**Sequence**<br>gabcdef gabcdef gabcdef gabcdef gabcde	**dTm (°C)**	**Target Heptad Binding**	**Library Heptad Binding**
**1**	QIDTLEA EIDQLED KNYALKT ELANLEK EIEKLE	53	1	1
**2**	QIDTLEA EIDQLED KNYALKT EIANLEK EIEKLE	52	1	1
**3**	KIDTLEA EIDQLED KNYALKT EIANLEK EIEKLE	51	1	1
**4**	KIDTLEA EIDQLED KNYALKT ELANLEK EIEKLE	51	1	1
**5**	KIDTLQA EIDQLED KNYALKT EIANLEK EIEKLE	49	1	1
**6**	QIDTLEA EIDQLED ENYALET EIANLEK EIEKLE	49	1	1
**7**	KIDTLQA EIDQLED KNYALKT ELANLEK EIEKLE	49	1	1
**8**	QIDTLEA EIDQLED ENYALET ELANLEK EIEKLE	49	1	1
**9**	KIDTLKA EIDQLED KNYALKT ELANLEK EIEKLQ	49	1	1
**10**	KIDTLEA EIDQLED KNYALKT ENANLEK EIEKLE	48	1	1
**11**	KIDTLEA EIDQLED KNYALKT EIANLE	8	1	1
**12**	KIDTLEA EIDQLED KNYALKT ELANLE	8	1	1
**13**	EIDQLED KNYALKT ELANLEK EIEKLQ	6	2	1
**14**	QIDTLEA EIDQLED KNYALKT ELANLE	6	1	1
**15**	QIDTLEA EIDQLED ENYALET ELANLE	6	1	1
**16**	QIDTLEA EIDQLED KNYALKT EIANLE	5	1	1

**17**	QIDTLEA EIDQLED ENYALET EIANLE	5	1	1
**18**	KIDTLQA EIDQLED KNYALKT ELANLE	5	1	1

isCAN Alternate Heptad Alignment

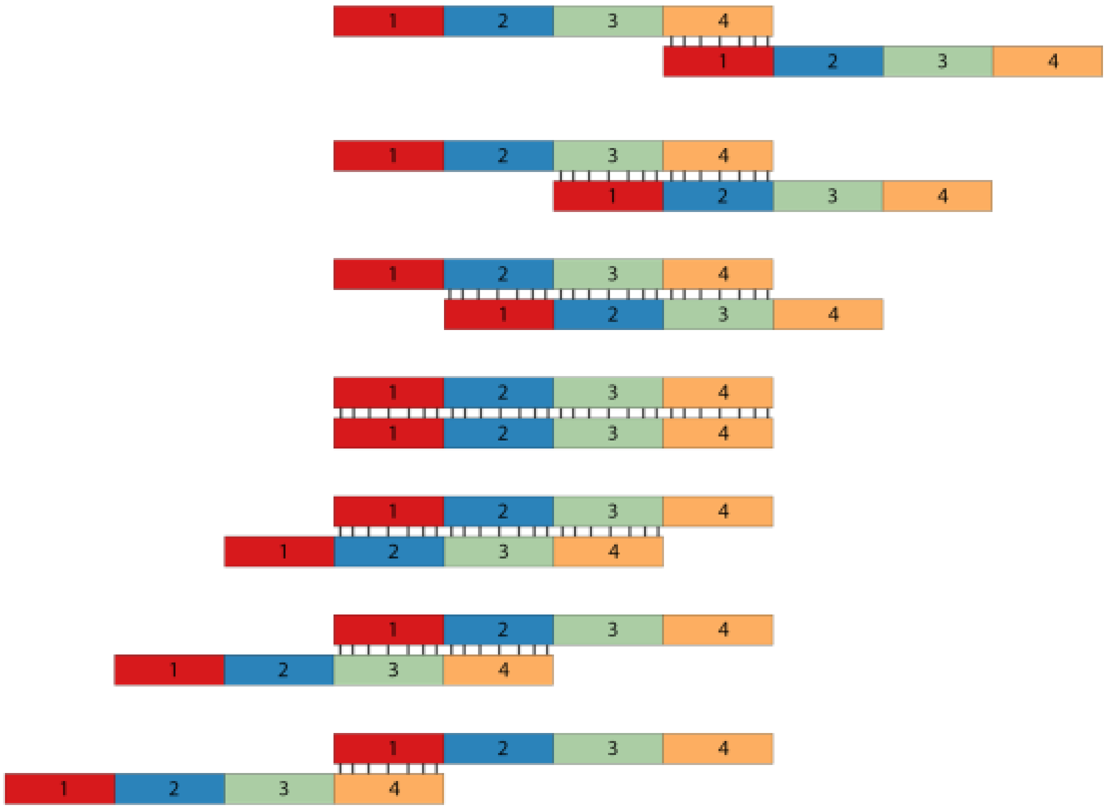

Supporting Figure 2 – Schematic of alternate heptad alignment. *During screening, the heptad register of the library is preserved while the peptide is systematically shifted along the target, one heptad at a time. All heptad alignments are evaluated and that which provides the most favourable* ΔT_m *is chosen.*

Supporting Table 14 – AlphaFold 3 statistics for isCAN hits from a library targeting cJun. *Models were generated using AlphaFold 3 [6] accessed via the AlphaFold Server (https://alphafoldserver.com/ [last accessed 02 November 2025]). Library sequences are from Supporting Table 13, hits 11 – 18. Models are in the same order as hits from Supporting Table 13, i.e. Model 1 corresponds to Hit 11, etc.*

Model	Target sequence	Library Sequence	Seed	ipTM	pTM	Target Heptad Binding	Library Heptad Binding
**1**	RIARLEEKVKTLKAQNSELASTANMLREQVAQLK	KIDTLEAEIDQLEDKNYALKTEIANLE	786574740	0.7	0.75	1	1
**2**	RIARLEEKVKTLKAQNSELASTANMLREQVAQLK	KIDTLEAEIDQLEDKNYALKTELANLE	1657184718	0.55	0.67	1	1
**3**	RIARLEEKVKTLKAQNSELASTANMLREQVAQLK	EIDQLEDKNYALKTELANLEKEIEKLQ	2120372269	0.74	0.79	2	1
**4**	RIARLEEKVKTLKAQNSELASTANMLREQVAQLK	QIDTLEAEIDQLEDKNYALKTELANLE	1017660287	0.6	0.7	1	1
**5**	RIARLEEKVKTLKAQNSELASTANMLREQVAQLK	QIDTLEAEIDQLEDENYALETELANLE	610707507	0.61	0.71	1	1
**6**	RIARLEEKVKTLKAQNSELASTANMLREQVAQLK	QIDTLEAEIDQLEDKNYALKTEIANLE	1843656129	0.74	0.78	1	1
**7**	RIARLEEKVKTLKAQNSELASTANMLREQVAQLK	QIDTLEAEIDQLEDENYALETEIANLE	916648553	0.79	0.8	1	1
**8**	RIARLEEKVKTLKAQNSELASTANMLREQVAQLK	KIDTLQAEIDQLEDKNYALKTELANLE	1465301849	0.59	0.69	1	1

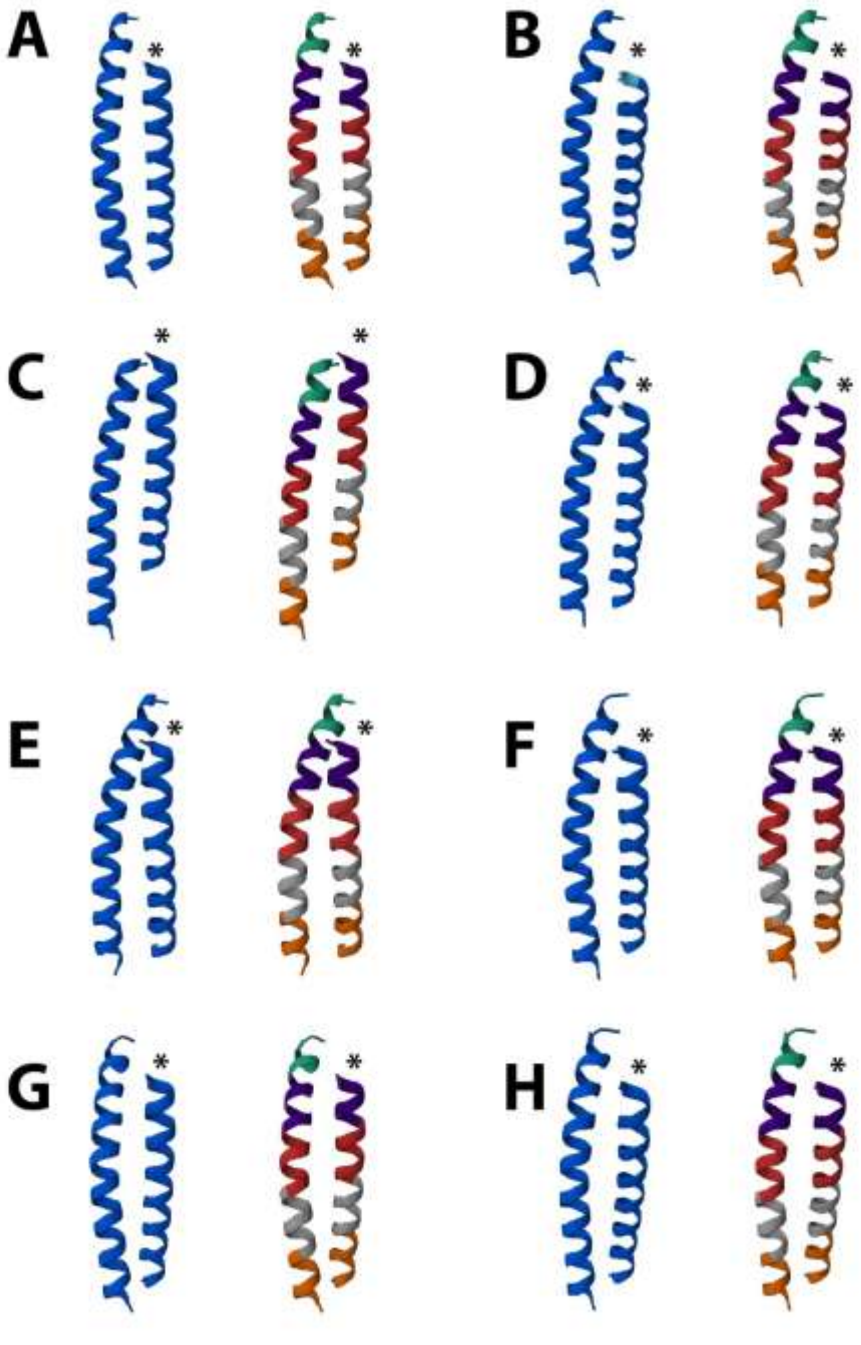

Supporting Figure 3 – AlphaFold 3[6] predicted models of isCAN hits targeting cJun. *AlphaFold 3 was accessed via AlphaFold 3 server (https://alphafoldserver.com/). Sequences and model statistics can be found in Supporting Table 14.* ***A – H*** *represent Models 1 – 8. The library sequences have been denoted with a *, Models are either coloured using plDTT confidence metrics (left) or according to heptad number (right). plDTT colour scheme: > 90 - dark blue, 90 > plDTT > 70 – cyan. Heptad colour scheme: heptad 1 – orange, heptad 2 – grey, heptad 3 – red, heptad 4 – purple, heptad 5 – green. It should be noted that heptad 5 in the target peptide (cJun) is only 6 residues long and lacks the terminal* ***f*** *residue. The same is true for heptad 4 in the library peptides, where it is only 6 residues long and lacks the terminal* ***f*** *residue. For all* ***A – H,*** *except* ***C****, the target – library binding is on heptads 1 – 4 across all peptides, whereas with* ***C,*** *the binding interface is heptads 2 – 5 on the target with heptads 1 – 4 on the library peptide.*

Automatic Heptad Frame Determination

The automatic heptad frame determination algorithm utilises an initial leucine-based search to identify putative ***d*** positions within the sequence. It does this by, first identifying all leucine residues, then searches for the longest series of leucines occurring at seven-residue intervals, which are provisionally assigned to the ***d*** position. These are subsequently used to identify the most likely heptad register.

At occasion, the leucine-based search does not yield a unique or reliable prediction. Our analyses indicate that inaccuracies arise primarily when multiple equally long series of leucines (each spaced seven residues apart) are identified. To prevent inaccurate predictions, we additionally discard the leucine-based search results when insufficient leucine residues are present to provide information for register inference.

In situations wherein the leucine-based search is inadequate, the algorithm invokes a secondary method for frame detection. It utilises the residue frequency table established by Lupas *et al*., [7] to score each residue across all possible heptad registers and assigns the sequence to the register with the highest cumulative score. When the leucine-based search identifies multiple plausible registers, residue scoring is weighted towards those candidates, whereas where the results of the leucine-based search are discarded, residue scoring is unweighted.

Validation

The algorithm was validated using two datasets. The first dataset utilised all 75 sequences available to access through InsiliCoil's sequence tables. 51 of these sequences were bZIP sequences from Seldeen *et al*.,[8] with an initial ***g*** residue added. 24 of these sequences are bHLHZIP sequences from Supporting Figure 1. All 75 sequences were ***g*** register. An additional 75 sequences were produced from the initial 75, by randomly trimming initial residues to generate sequence variants of differing registers, for a total of 150 sequences. The 75 sequences generated by trimming varied on each validation. Across five validations of this dataset, the automatic heptad frame determination algorithm accurately determined 100 % of heptad registers with an average time of 42.67 milliseconds per validation.

The second dataset used is based on the cFos-based library from Lathbridge *et al*.,[2] (Supporting Table 3). The library used has 60,466,176 sequences all ***g*** register. Each sequence was duplicated 14 times, with each having between 0 and 13 initial residues trimmed. This resulted in a total of 846,526,464 sequences, with 120,932,352 sequences per heptad register. Across three validations of this dataset, the automatic heptad determination algorithm determined 100 % of heptad registers with an average time of 49.25 minutes per validation.

References

(1) Madeira, F.; Madhusoodanan, N.; Lee, J.; Eusebi, A.; Niewielska, A.; Tivey, A. R. N.; Lopez, R.; Butcher, S. The EMBL-EBI Job Dispatcher sequence analysis tools framework in 2024. *Nucleic Acids Research* **2024**, *52* (W1), W521-W525. DOI: 10.1093/nar/gkae241.

(2) Lathbridge, A.; Mason, J. M. Computational Competitive and Negative Design To Derive a Specific cJun Antagonist. *Biochemistry* **2018**, *57* (42), 6108-6118. DOI: 10.1021/acs.biochem.8b00782.

(3) Yu, M.; Tang, T. M. S.; Ghamsari, L.; Yuen, G.; Scuoppo, C.; Rotolo, J. A.; Kappel, B. J.; Mason, J. M. Exponential Combination of a and e/g Intracellular Peptide Libraries Identifies a Selective ATF3 Inhibitor. *ACS Chemical Biology* **2024**, *19* (3), 753-762. DOI: 10.1021/acschembio.3c00779.

(4) Crooks, R. O.; Lathbridge, A.; Panek, A. S.; Mason, J. M. Computational Prediction and Design for Creating Iteratively Larger Heterospecific Coiled Coil Sets. *Biochemistry* **2017**, *56* (11), 1573-1584. DOI: 10.1021/acs.biochem.7b00047.

(5) Gradišar, H.; Jerala, R. De novo design of orthogonal peptide pairs forming parallel coiled-coil heterodimers. *Journal of Peptide Science* **2011**, *17* (2), 100-106. DOI: 10.1002/psc.1331.

(6) Abramson, J.; Adler, J.; Dunger, J.; Evans, R.; Green, T.; Pritzel, A.; Ronneberger, O.; Willmore, L.; Ballard, A. J.; Bambrick, J.; et al. Accurate structure prediction of biomolecular interactions with AlphaFold 3. *Nature* **2024**, *630* (8016), 493-500. DOI: 10.1038/s41586-024-07487-w.

(7) Lupas, A.; Van Dyke, M.; Stock, J. Predicting Coiled Coils from Protein Sequences. *Science* **1991**, *252* (5009), 1162-1164. DOI: 10.1126/science.252.5009.1162.

(8) Seldeen, K. L.; Deegan, B. J.; Bhat, V.; Mikles, D. C.; McDonald, C. B.; Farooq, A. Energetic coupling along an allosteric communication channel drives the binding of Jun-Fos heterodimeric transcription factor to DNA. *The FEBS Journal* **2011,** *278* (12), 2090-2104. DOI: 10.1111/j.1742-4658.2011.08124.x.
